# Development and validation of an epigenetic signature of allostatic load

**DOI:** 10.1042/BSR20241663

**Published:** 2025-04-09

**Authors:** Jonviea D. Chamberlain, Daniel Ackermann, Murielle Bochud, Tom Booth, Laurence Chapatte, Janie Corley, Simon R. Cox, Sarah E. Harris, Cassandre Kinnaer, Robert-Paul Juster, Isabella Locatelli, David Nanchen, Belène Ponte, Menno Pruijm, Sylvain Pradervand, Paul G. Shiels, Silvia Stringhini, Sébastien Nusslé, Semira Gonseth-Nusslé

**Affiliations:** 1 Department of Epidemiology and Health Systems (DESS), Unisanté, University Center for Primary Care and Public Health, Lausanne, Switzerland; 2 Department for Nephrology and Hypertension Inselspital, Inselspital, Bern University Hospital and University of Bern, Bern 3010, Switzerland; 3 Lothian Birth Cohorts, Department of Psychology, University of Edinburgh, Edinburgh, UK; 4 Genknowme, Epalinges, Switzerland; 5 Department of Psychology, University of Montreal, Montreal, Quebec, Canada; 6 Center on Sex∗Gender, Allostasis, and Resilience (CESAR), Montreal Mental Health University Institute, Montreal, Quebec, Canada; 7 Department of Health Promotion and Prevention (DPSP), Unisanté, University Center for Primary Care and Public Health, Lausanne, Switzerland; 8 Department of Nephrology and Hypertension, Geneva University Hospitals, Geneva, Switzerland; 9 Service of Nephrology and Hypertension, University Hospital of Lausanne, and University of Lausanne, Lausanne 1011, Switzerland; 10 Vital-IT Group, Swiss Institute of Bioinformatics, Lausanne, Switzerland; 11 Genomic Technologies Facility, University of Lausanne, Lausanne, Switzerland; 12 School of Molecular Biosciences, Davidson Building, MVLS, University of Glasgow, Glasgow G12 8QQ, UK; 13 Department of Epidemiology and Health Systems (DESS), Unisanté, University Center for Primary Care and Public Health, Lausanne, Switzerland; 14 Unit of Population Epidemiology, Division of Primary Care Medicine, Geneva University Hospitals & Faculty of Medicine, University of Geneva, Geneva, Switzerland

**Keywords:** Allostatic load, DNA methylation, Chronic Stress, Epigenetic signature, Prevention, Chronic diseases

## Abstract

The allostatic load (AL) concept measures physiological dysregulation in response to internal and external stressors that accumulate across the life course. AL has been consistently linked to chronic disease risk across studies. However, there is considerable variation in its operationalization. In the present study, DNA methylation (DNAm) data (using the Illumina Infinium MethylationEPIC BeadChip array) from the Swiss Kidney Project on Genes in Hypertension (SKIPOGH) cohort, a Swiss-based family cohort study, were used in a discovery epigenome-wide association study to identify cytosine–guanine nucleotide sites associated with phenotypic measures of AL. Elastic net linear regression models were used to estimate an epigenetic signature of AL (methAL), including an Illumina HumanMethylation450K (HM450K) assay-compatible signature (methALT). The methALT signature was validated in the 1936 Lothian Birth Cohort (LBC1936), population-based prospective cohort study. We found that the methAL signature was positively associated with the clinical phenotype of AL in both the SKIPOGH (*R*^2^ = 0.59) and LBC1936 (*R*^2^ = 0.16) cohorts. In the validation cohort, a one standard deviation increase in methALT signature was associated with 25% higher odds of reported history of cardiovascular disease (CVD) (odd ratio [OR] = 1.25, 95% confidence interval [CI] = 1.05–1.50), and a nearly two-fold increase in all-cause mortality rate at the beginning of follow-up (hazard ratio = 1.68, 95% CI = 1.33–2.13) when adjusting for all potential confounders. In conclusion, the epigenetic signature for AL not only correlated well with phenotype-based AL scores but also exhibited a stronger association with the history of CVD and all-cause mortality compared with AL scores. The methAL signature could help assuage issues of comparison across studies.

## Introduction

Understanding what underpins healthy aging, or ‘the process of developing and maintaining the functional ability that enables wellbeing in older age’ (WHO definition), is essential for reducing the global burden of diseases. Allostatic load (AL) represents a theoretical and evidence-backed concept that measures the physiological dysregulation of the body and brain in response to internal and external stressors that accumulate across the life course (i.e. the ‘wear-and-tear’) as we age by integrating different biomarkers [1]. Conceptually, AL bridges environmental exposures, lifestyle exposures, and disease as part of the human life course exposome and is one of the most comprehensive models of chronic stress.

While evidence for the link between AL and chronic disease has been relatively consistent across studies over the last 30 years, there is considerable variation in its operationalization [2,3]. Variation across studies includes the differences in the biomarkers used, the summarization method used to create a single score of AL, and the definition of high-risk [4,5]. For example, although the original proposed score included 10 biomarkers – for the cardiovascular, metabolic, neuroendocrine, and immune systems – a recent systematic review stated that the number of reported biomarkers varied between 6 and 24, with some papers excluding certain system-specific biomarkers [1,4]. A second difficulty of operationalizing AL is the influence of the population structure on risk categorization for each AL biomarker. For example, C-reactive protein (CRP) levels change with age, with CRP levels in the elderly being generally higher compared with younger ages [6].

Epigenetic modifications can be induced in response to external (i.e. environmental exposures) or internal stimuli, resulting in differences in gene expression. Epigenetic modifications regulate chromatin structure and DNA accessibility – and thereby gene expression – via histone modifications, DNA methylation (DNAm), or non-coding RNAs. DNAm, the focus of the present manuscript, results from the addition of a methyl group at cytosine–guanine nucleotides (CpGs). Epigenetic signatures of DNAm can summarize the epigenetic modifications that occur at different loci and regions of DNA in association with a phenotype of interest, for example, lifestyle exposures such as tobacco or alcohol consumption [7]. In response to the operationalization issues mentioned above, an epigenetic signature of AL could help to circumvent measurement issues and offer a standardized approach. To date, no study has identified differential methylation in association with AL nor estimated a replicable signature of AL. Therefore, the objective of this study was to present and validate an epigenetic signature of AL.

## Methods

### Cohorts

The Swiss Kidney Project on Genes in Hypertension (SKIPOGH) is a Swiss family-based population cohort with a baseline and follow-up visit. Participants were recruited between December 2009 and April 2013 for the baseline examination [8]. Individuals were eligible for inclusion if they were (a) 18 years or older, (b) self-identified as White, (c) had at least one first-degree family member willing to participate, and (d) provided written consent for study inclusion. Clinical biomarkers were collected at one of three study centers, and standardized questionnaires were filled out by the participants at home. Whole blood samples were collected at baseline and the follow-up survey (October 2012–December 2016). Prevalent cases of cardiovascular disease (CVD) (yes/no) were determined according to participants’ response of having a history of CVD-related events (e.g. coronary heart disease, stroke, or any other cardiovascular-related events such as coronary disease, angina, and infarct).

The 1936 Lothian Birth Cohort (LBC1936) is a longitudinal, population-based cohort study including participants from the original 1947 Scottish Mental Survey. Participants completed an interview administered by a trained psychologist and research nurse, which included sociodemographic characteristics, cognitive, physical, and other assessments including blood sampling (for DNA extraction and clinical biomarker assessment), as well as a follow-up at-home questionnaire that covered topics such as experiences, activity levels, personality-related traits, life satisfaction, social support, and food frequency questionnaires [9]. Included participants were then followed at approximately 3-year intervals for a total of six waves, with clinical biomarkers collected across all study waves. Cardiovascular disease status was defined as a dichotomous variable based on the question, ‘Have you ever had a heart attack, angina, heart valve problem, abnormal heart rhythm, or any other heart problem?’. Vital status was assessed by linkage to the National Health Service Central Register in Scotland as of January 2022. The present study uses clinical biomarkers and DNAm data collected during the first wave of follow-up.

### DNAm

In the SKIPOGH cohort, whole blood samples were collected and stored at −80°C. DNAm was measured by extracting DNA from white blood cells using the KingFisher Duo robot extraction system (ThermoFisher, Waltham, Massachusetts), denaturing DNA (~1.2 μg) with sodium bisulfate using the EZ DNA Methylation© Kit (Zymo Research), and then amplifying the DNA by polymerase chain reaction using the Illumina Infinium© Methylation Assay, resulting in a final elution of 8 µl of M-Elution Buffer. For 250 SKIPOGH participants, DNAm levels were assayed using the Illumina Infinium HumanMethylation450 BeadChip, while for the remaining 736 participants, DNAm levels were assayed using the Illumina Infinium MethylationEPIC BeadChip (EPIC). Methylation data were normalized using an adapted version of the CPACOR pipeline where reference distributions of signal intensities from eight reference samples are computed and upon which signal intensities from the entire cohort are quantile normalized (functions ‘normalize.quantiles.determine.target’ and ‘normalize.quantiles.use.target’ from R library ‘preprocessCore’) [10,11]. Beta values were calculated using the normalized data.

Methylation data for the LBC1936 cohort were processed using an Illumina HumanMethylation450K array (HM450K) using DNA extracted from whole blood samples [12]. Methylation data were similarly normalized according to separate internal controls.

### Clinical phenotype for allostatic load

#### SKIPOGH cohort

The clinical phenotype of AL (please refer to Table 1 for a list of included biomarkers) was operationalized using multiple methodologies [13], including

1. a weighted sum-score based on risk as defined in the study population,
2. a sum-score of absolute z-score values (AL-Z),
3. sum-score of risk according to general population cut-off values (AL-GP), and
4. latent variables for each AL system (i.e. inflammatory, metabolic, cardiovascular, and neuroendocrine).

**Table 1:** Biomarkers included in phenotype-based measures of allostatic load

	SKIPOGH		LBC1936
	Weighted sum	Latent variable	
*Neuroendocrine system*			
Cortisol (μg), 24 hours	X	X	
Androsterone (μg), 24 hours	X	X	
Dehydroepiandrosterone (μg), 24 hours	X	X	
Self-reported stress^*^		X	
*Inflammatory system*			
CRP (mg/l)	X	X	X
IFN-γ (pg/ml)	X	X	
TNFa (pg/ml)	X	X	
IL-10 (pg/ml)	X	X	
IL-6 (pg/ml)	X	X	
IL-1b (pg/ml)	X		
Fibrinogen			X
*Metabolic system*			
Total cholesterol, blood (mmol/l)	X		
Total cholesterol/HDL ratio			X
GGT, blood (U/l)	X	X	
Glucose, blood (mmol/l)	X	X	x
Insulin, blood (mU/l)	X	X	
Low-density lipoprotein	X		
Blood triglyceride (mmol/l)	X		X
ALAT (U/l)	X	X	
Albumin (g/l)	X	X	X
Blood HDL cholesterol (mmol/l)	X		
Lipoprotein(a) (mg/ml)	X		
Uric acid (μmol/l)	X	X	
*Cardiovascular system*			
Systolic blood pressure, mean	X	X	X
Diastolic blood pressure, mean	X	X	X
Heart rate, mean	X	X	
*Anthropometric*			
Body mass index (kg/m^2^)	X	X	X
Waist-to-hip ratio	X	X	
Body surface area (DuBois & DuBois)		X	

* Self-reported stress defined as ‘On a scale from 1 to 10, what is your level of daily stress’?

CRP, C-reactive protein . IFN-γ, interferon gamma. ALAT, alanine transaminase. TNF-α, tumor necrosis factor-α. IL, interleukin. GGT, gamma-glutamyl tranferase. HDL, high-density lipoprotein. SKIPOGH, Swiss Kidney Project on Genes in Hypertension. LBC1936, Lothian Birth Cohort 1936. AL, allostatic load.

This initial operationalization of AL according to multiple definitions was performed to consider the numerous AL formulations used in extant AL literature [14]. For the first definition of AL – the weighted sum score – risk (0/1) was determined based on the distribution of each individual biomarker within the SKIPOGH population; such that, individuals were classified as ‘at risk’ if they fell within the lower or upper quartile of the distribution (biomarker-dependent). Risk scores were then summed together within each system and divided by the total number of system-specific biomarkers. The system-specific scores were then summed together to create the final unit-weighted sum score ranging from 0 to 4. For the second definition (2) of AL, the absolute z-score values for nine different biomarkers were summed together. The third definition (3) categorized risk (0/1) according to cut-off values identified in extant literature (Table 1) [15]. Risk scores were then summed together to create an unweighted sum score of AL score ranging from 0 to 14 (AL-GP). Finally, latent variables were estimated for each system separately (i.e. neuroendocrine, cardiovascular, immune, and metabolic) using an exploratory factor analysis that included scaled biomarkers for each system (Table 1) [16]. Latent variables were constructed based on maximum likelihood, unidirectional factor loadings of included clinical biomarkers, root mean square error of approximation (<0.1), and the Comparative Fit Index (>0.90). The ‘lavPredict’ function was used to estimate values for each individual latent variable. Factor loadings for each latent variable are provided in Supplementary Figures S1–S4.

#### LBC1936 cohort

AL in the LBC1936 cohort was calculated as the sum of the absolute z-scores for nine different biomarkers, according to previously published scores using LBC data (Table 1) [17]. Prior to z-score calculation, the distributions of CRP, HBA1c, and triglycerides were normalized using a box-Cox transformation. Z-scores for the total cholesterol to high-density lipoprotein ratio were converted so that values below zero were transformed to zero prior to AL calculation. Medications were characterized according to the potential influence on AL (yes/no), particularly in relation to inflammation.

### Statistical analyses

Missing data for the clinical biomarkers of AL were assumed to be missing at random and imputed using an iterative, random forest method available in the R package missForest [18]. To verify that imputed values did not meaningfully affect results, sensitivity analyses were carried out using non-imputed data in the validation cohort.

#### Epigenome-wide association study

Epigenome-wide association studies (EWASs) were carried out restricting to EPIC-based methylation data from the SKIPOGH population (*N* = 737). Separate EWASs were run for each AL phenotype as described above, and individual latent variables. A linear mixed model was run for each CpG site (β) as follows:

*β* = AL phenotype + sex + age (at the time of blood sample) + blood cell counts + technical covariates (30 PCs) + familial structure

CpGs were selected for validation based on a Bonferroni-adjusted threshold (*α* = 10^−7^). Manhattan plots and Volcano plots are provided in the Supplementary Material.

#### Epigenetic signature

The SKIPOGH data were divided into a training (*N* = 500) and validation set (*N* = 236) based on ID sequence, again restricting to participants with EPIC-based methylation data. To estimate the epigenetic signature of AL, CpGs identified in EWASs were grouped together into a list of candidate CpGs. Relevant CpGs in association with AL – according to definition (1) as described above – were then selected using an elastic-net linear regression model considering combinations of tuning parameters ɑ and λ [19]. The AL score based on GP population cut-offs was chosen for the creation of the epigenetic signature to ensure a signature generalizable to all cohorts, as opposed to older-aged or disease-specific cohorts. The intercept and coefficient values for CpGs identified from the model with the smallest prediction error (mean square error) based on combinations of ɑ and λ were used for signature creation. Two signatures were created to ensure interoperability with both EPIC and HM450k methylation platforms:

1. methAL: including CpGs available on the EPIC platform;
2. methALT: including only those CpGs available on both EPIC and HM450K platforms.

#### Association with disease and mortality

Logistic regression models were used to assess the association between AL and the history of cardiovascular disease – presented as odds ratios (ORs) with 95% confidence intervals (CIs) – using both SKIPOGH and LBC1936 data. Fully adjusted models were adjusted for age at baseline (continuous variable), sex (dichotomized variable), educational attainment (SKIPOGH: categorical variable low, medium, and high; LBC1946: continuous variable, years of education), body mass index (continuous variable), and tobacco consumption (continuous variable, measured via the epigenetic signature, epiTob [7]). To assess the association between AL and risk of mortality, only possible in the LBC1936 cohort, a Cox regression model was used to estimate hazard ratios (HRs) and associated 95% CIs. The underlying time scale was the time since study entry. Proportional hazards were assessed using scaled Schoenfeld residuals in correlation with time using the *cox.zph* function of the *survival* package [20]. Non-proportional hazards were addressed by the creation of a person–period data structure and the inclusion of an interaction between signature and time. In this manner, data were transformed into a long format, such that for each included participant, the follow-up time is split at each unique event time so that each row corresponds to a unique follow-up period, at the end of which the event of interest occurs or not (i.e. mortality). To facilitate direct comparisons across variations of AL operationalizations, all regression results are presented as standardized values where a one-unit increase corresponds to an increase in one standard deviation.

## Results

Population characteristics for the SKIPOGH (*N* = 1,034) and LBC1936 (*N* = 1,091) cohorts are presented in Table 2. The SKIPOGH cohort was younger, had a lower average BMI, and had lower levels of AL-associated clinical biomarkers in comparison with the LBC1936 cohort (Table 2). Epigenetic data were available for 976 SKIPOGH participants and 895 LBC1936 participants. Among those included in the analytic sample, 15.2% of SKIPOGH participants (*N* = 148) and 24.4% of LBC1936 participants (*N* = 218) reported a history of CVD; 376 LBC1936 participants died during follow-up, resulting in 12,149.1 years of follow-up time.

**Table 2:** Descriptive statistics for the SKIPOGH and LBC1936 cohorts stratified by sex

*Variable*	SKIPOGH				LBC1936		
	Male (*N* = 486)	Female (*N* = 548)		*P* value	Male (*N* = 548)	Female (*N* = 543)	*P* value
Age (years)				0.795			0.883
Mean (SD)	50.43 (17.51)	50.70 (17.08)			69.72 (0.83)	69.73 (0.84)	
Q1, Q3	35.00, 65.40	36.10, 63.35			69.07, 70.42	69.10, 70.42	
Education^1^				0.032			0.783
High/Mean (SD)	174 (38.0%)	185 (36.4%)			10.75 (1.16)	10.73 (1.10)	
Middle/Q1, Q3	216 (47.2%)	215 (42.3%)			10.00, 12.00	10.00, 11.50	
Low	68 (14.8%)	108 (21.3%)					
N-miss	28	40					
Body mass index (kg/m^−2^)				<0.001			0.055
Mean (SD)	26.31 (4.27)	24.43 (4.75)			28.03 (3.88)	27.53 (4.77)	
Q1, Q3	23.41, 28.49	20.96, 26.90			25.27, 30.53	24.28, 30.07	
N-Miss	3	2			2	0	
Blood CRP				0.589			0.572
Mean (SD)	2.13 (4.91)	2.29 (4.63)			5.15 (6.56)	5.38 (6.79)	
Q1, Q3	0.50, 2.00	0.50, 2.00			1.50, 6.00	1.50, 6.00	
N-Miss	2	5			19	18	
Triglycerides				< 0.001			0.150
Mean (SD)	1.16 (0.75)	0.93 (0.47)			1.69 (0.83)	1.61 (0.99)	
Q1, Q3	0.69, 1.45	0.60, 1.14			1.13, 2.03	1.07, 1.92	
N-Miss	2	5			64	62	
Blood Hba1c				< 0.001			0.122
Mean (SD)	5.31 (1.18)	4.93 (1.03)			5.78 (0.73)	5.71 (0.56)	
Q1, Q3	4.80, 5.50	4.50, 5.20			5.40, 5.90	5.40, 5.80	
N-Miss	2	4			118	147	
Average SBP				<0.001			0.235
Mean (SD)	121.64 (14.24)	114.21 (16.07)			150.30 (19.20)	148.93 (18.88)	
Q1, Q3	111.20, 129.20	102.00, 124.00			137.00, 161.67	136.33, 161.33	
N-Miss	2	1			3	7	
Average DBP				<0.001			0.010
Mean (SD)	76.50 (8.84)	71.65 (8.62)			82.04 (10.28)	80.46 (9.93)	
Q1, Q3	70.80, 81.20	65.20, 78.00			74.67, 89.00	73.67, 87.08	
N-Miss	2	1			3	7	
Blood albumin				0.123			<0.001
Mean (SD)	40.49 (3.82)	40.12 (3.85)			45.08 (3.16)	44.25 (2.98)	
Q1, Q3	38.00, 43.00	37.00, 43.00			43.00, 47.00	42.00, 46.00	
N-Miss	2	4			17	15	
Blood fibrin							0.070
Mean (SD)					3.24 (0.64)	3.31 (0.64)	
Q1, Q3					2.80, 3.60	2.90, 3.60	
N-Miss					16	23	
HDL ratio				<0.001			0.043
Mean (SD)	2.54 (1.01)	1.98 (0.84)			3.83 (1.09)	3.69 (1.05)	
Q1, Q3	1.85, 3.17	1.39, 2.41			3.10, 4.50	3.00, 4.30	
N-Miss	179	227			65	60	
Tobacco signature				<0.001			0.008
Mean (SD)	−1.61 (2.74)	−2.53 (2.23)			1.04 (2.79)	0.55 (2.71)	
Q1, Q3	−3.27,–0.61	−3.67,–1.68			−0.91, 2.43	−1.14, 1.64	
N-Miss	21	47			95	101	
methAL				<0.001			
Mean (SD)	1.51 (0.54)	1.98 (0.76)					
Q1, Q3	1.13, 1.79	1.44, 2.36					
N-Miss	165	137					
methALT				<0.001			<0.001
Mean (SD)	1.07 (0.91)	1.61 (1.05)			3.41 (0.87)	2.57 (0.80)	
Q1, Q3	0.67, 1.64	1.00, 2.28			2.79, 3.99	2.00, 3.04	
N-Miss	47	22			95	101	

Only AL-related phenotypes that were used to create composite score for the LBC1936 cohort are included.

1 For the LBC1936 cohort, education was included as a continuous variable, i.e. years of education.

CRP, C-reactive protein. SBP, systolic blood pressure. HDL, high-density lipoprotein. SKIPOGH, Swiss Kidney Project on Genes in Hypertension. LBC1936, Lothian Birth Cohort 1936. AL, allostatic load. DBP, Diastolic blood pressure.

In the discovery EWAS, 23 CpGs were associated with the clinical AL-GP phenotype (Bonferroni *P* value < 5.77^-8) and 5 CpGs for the AL-Z phenotype (Supplementary Figure S1). Latent variable construction and factor loadings are provided in Supplementary Figures S2–S5. The EWASs of latent variables resulted in the identification of 3 CpGs in association with the cardiovascular system, 20 for the metabolic system, 186 for the inflammatory system, and 192 for the neuroendocrine system (Supplementary Figure S6).

### Epigenetic signature creation and validation

A final selection of 32 CpG sites were included in the methAL signature, and 23 CpGs in the methALT signature (Supplementary Tables S1 and S2). The methAL signature included CpGs across all systems (as identified with latent variables).

**Figure 1:**
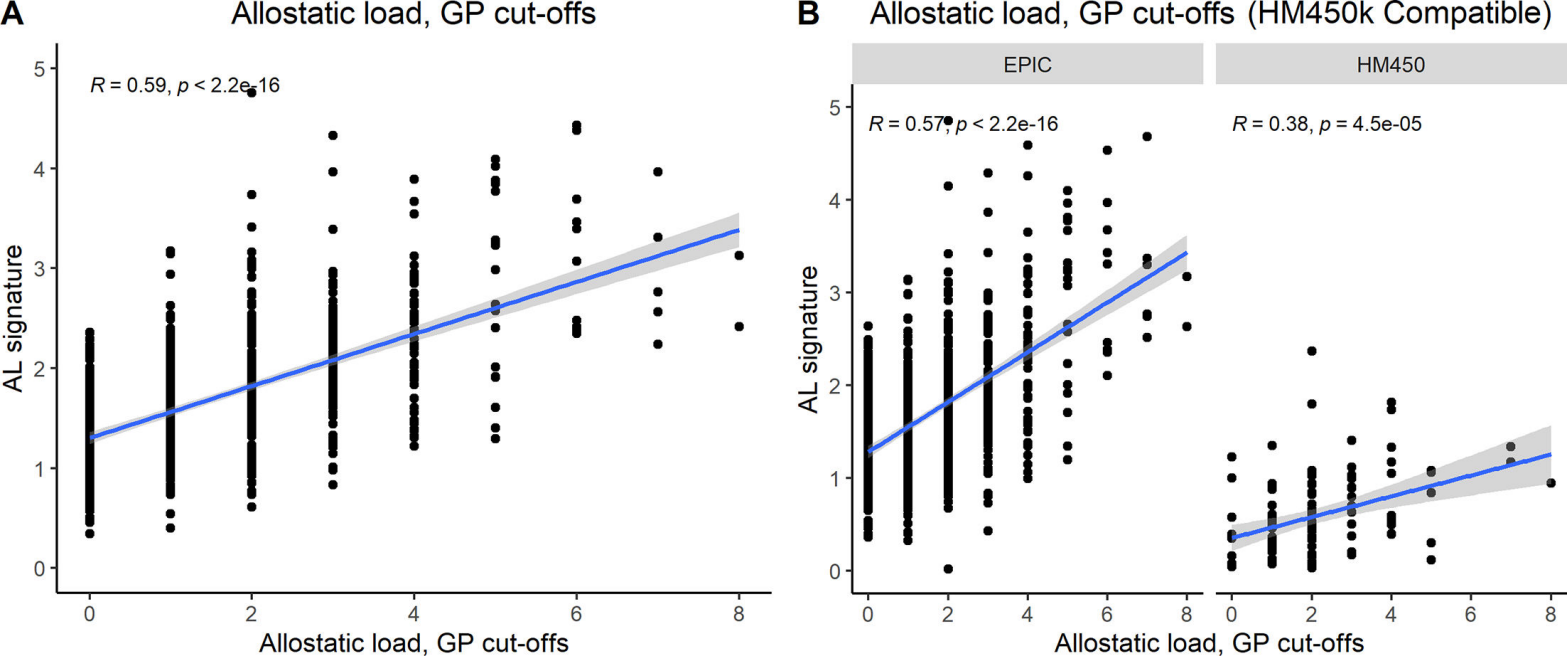
Plot of the correlation between phenotypic allostatic load and the epigenetic signature in the SKIPOGH cohort. (**A**). The correlation between AL-GP (the phenotype-based score) and the methAL signature. (**B**). The correlation between AL-GP and the HM-450K compatible methALT signature, stratified by sex. Plots were created using data from the SKIPOGH cohort.SKIPOGH, Swiss Kidney Project on Genes in Hypertension.

The correlation between the clinical AL phenotype and DNAm-based measures of AL is presented in Figure 1. Among all SKIPOGH participants, the methAL signature was positively associated with the clinical AL phenotype (*R^2^* = 0.59; *P* < 0.001) (Figure 1). Correlation coefficients for the methALT signature were attenuated when using only DNAm data from the HM450K platform. High correlations were observed between the EPIC-based DNAm signature and HM450K-compatible signature (Supplementary Figure S7). In the LBC1936 validation cohort, the methALT signature was correlated with the AL score (*R^2^* = 0.16, *P* value < 0.001) (Supplementary Figure S8).

#### MethAL and association with cardiovascular disease

**Figure 2:**
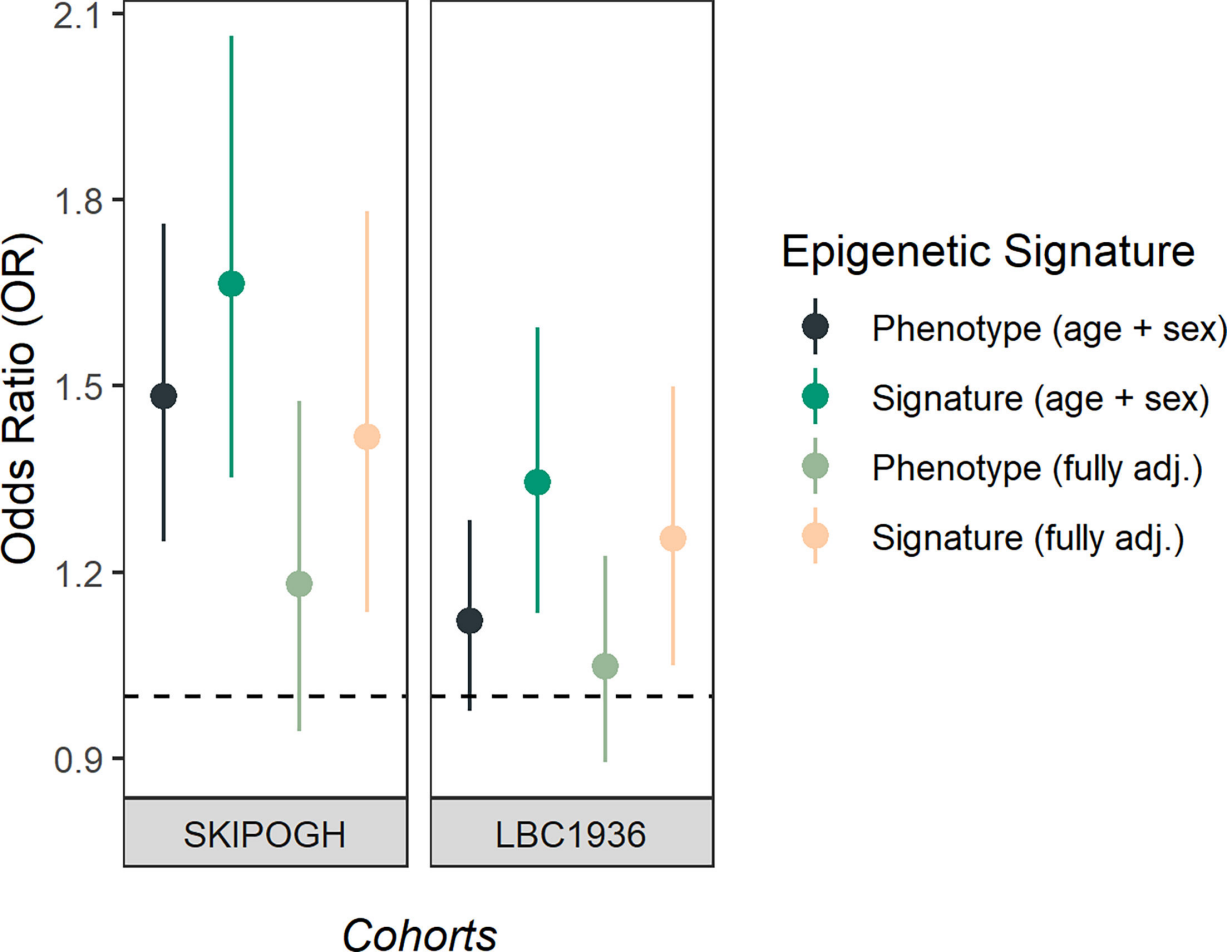
Plot of odds ratios and 95% confidence intervals for probability of history of cardiovascular disease corresponding to different measures of allostatic load. Odds ratios and 95% confidence intervals for the association between measures of AL and a history of cardiovascular disease. Presented odds ratios correspond to standardized signatures.

A comparison of phenotypic-based measures of AL with epigenetic signatures in association with CVD is presented in Table 3. Both the clinical phenotype of AL and the epigenetic signatures (methAL and methALT) were associated with a higher odds of reported CVD history in the SKIPOGH cohort (adjusting for age and sex).For example, a one-unit increase in AL-GP was associated with a nearly 50% increased odds of CVD history (OR = 1.48, 95% CI = 1.25–1.76), while a one-unit increase in methAL was associated with a two times higher odds of CVD history (OR = 2.00, 95% CI = 1.58–2.56). Following adjustment for additional confounders – including years of education, BMI, and tobacco consumption – the association between phenotypic estimates of AL and a history of CVD was attenuated and no longer statistically significant (*P* > 0.05) (Figure 2; Table 3). Sensitivity analyses on LBC1936 data excluding imputed data did not result in any meaningful change in the effect size of the association clinical AL scores at baseline and odds of CVD.

**Table 3:** Association between measures of allostatic load and history of cardiovascular disease

	SKIPOGH		LBC1936	
	Model A	Model B	Model A	Model B
Phenotype-based score				
AL^1^	1.48 (1.25–1.76)	1.18 (0.94–1.48)	1.12 (0.98–1.28)	1.05 (0.89–1.23)
Epigenetic signature				
methAL	2.00 (1.58–2.56)	1.80 (1.39–2.37)	..	..
methALT	1.67 (1.35–2.07)	1.42 (1.14–1.78)	1.34 (1.14–1.59)	1.25 (1.05–1.50)

This table presents odds ratios from a logistic regression model. Measures of AL are standardized. Model A is adjusted for age at study entry and sex; Model B is adjusted for age at entry, EpiTob signature, BMI, and years of education.

1 AL for the LBC cohort is based on the z-score method.

SKIPOGH, Swiss Kidney Project on Genes in Hypertension. LBC1936, Lothian Birth Cohort 1936. AL, allostatic load.

When using data from the LBC1936 cohort, no association was observed between the phenotypic measure of AL and the history of CVD after adjusting for all potential, known confounders. In contrast, the methALT signature was associated with CVD history (OR = 1.25, 95% CI = 1.05–1.50).

#### MethAL and risk of mortality

**Figure 3:**
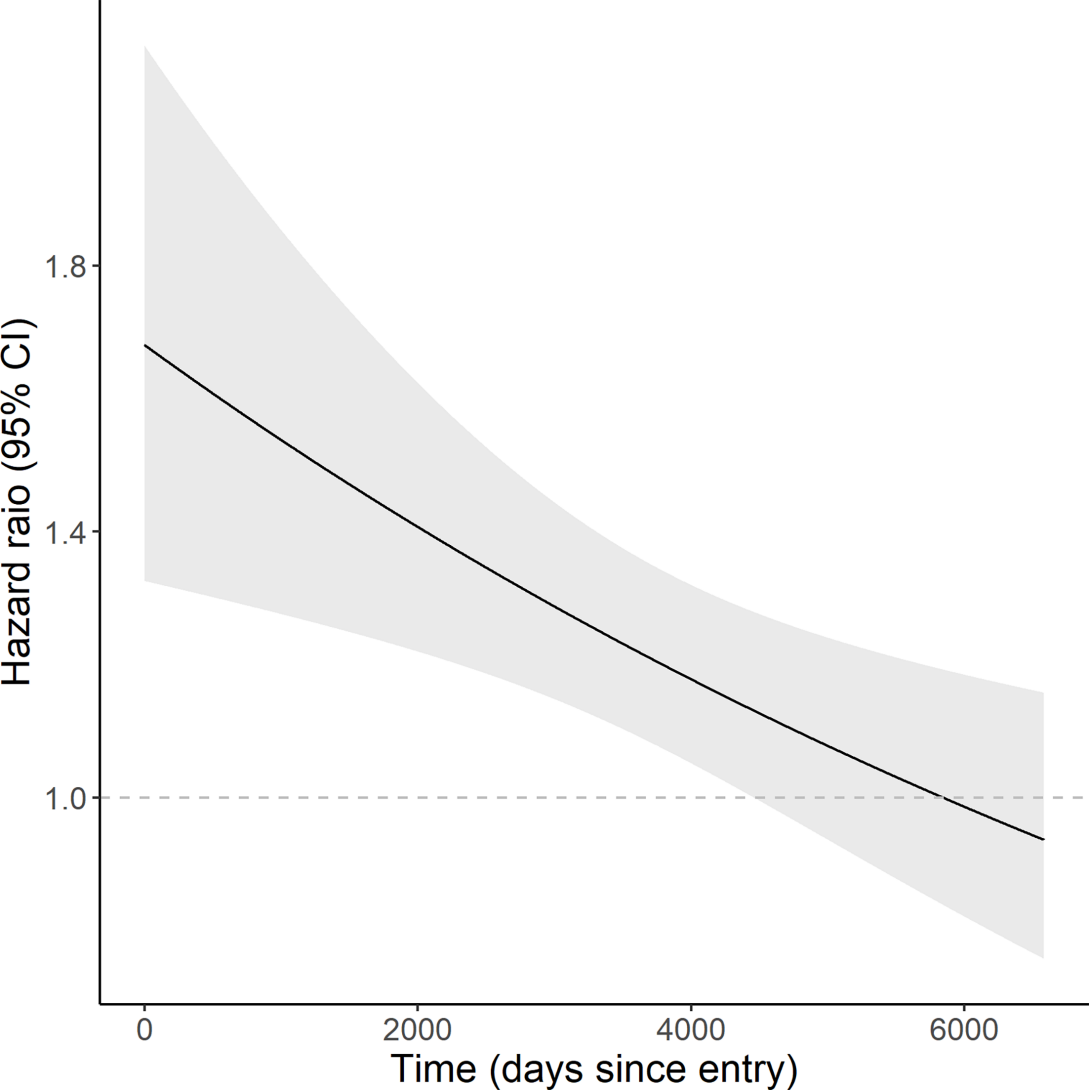
Association between methALT and the risk of mortality in the LBC1936 cohort over time. The solid black line represents the hazard ratio (HR) of the association between the methALT signature and risk of mortality with increasing time since study entry. HRs correspond to the standardized methALT signature. The gray area represents the 95% confidence interval. LBC1936, Lothian Birth Cohort 1936.

**Figure 4:**
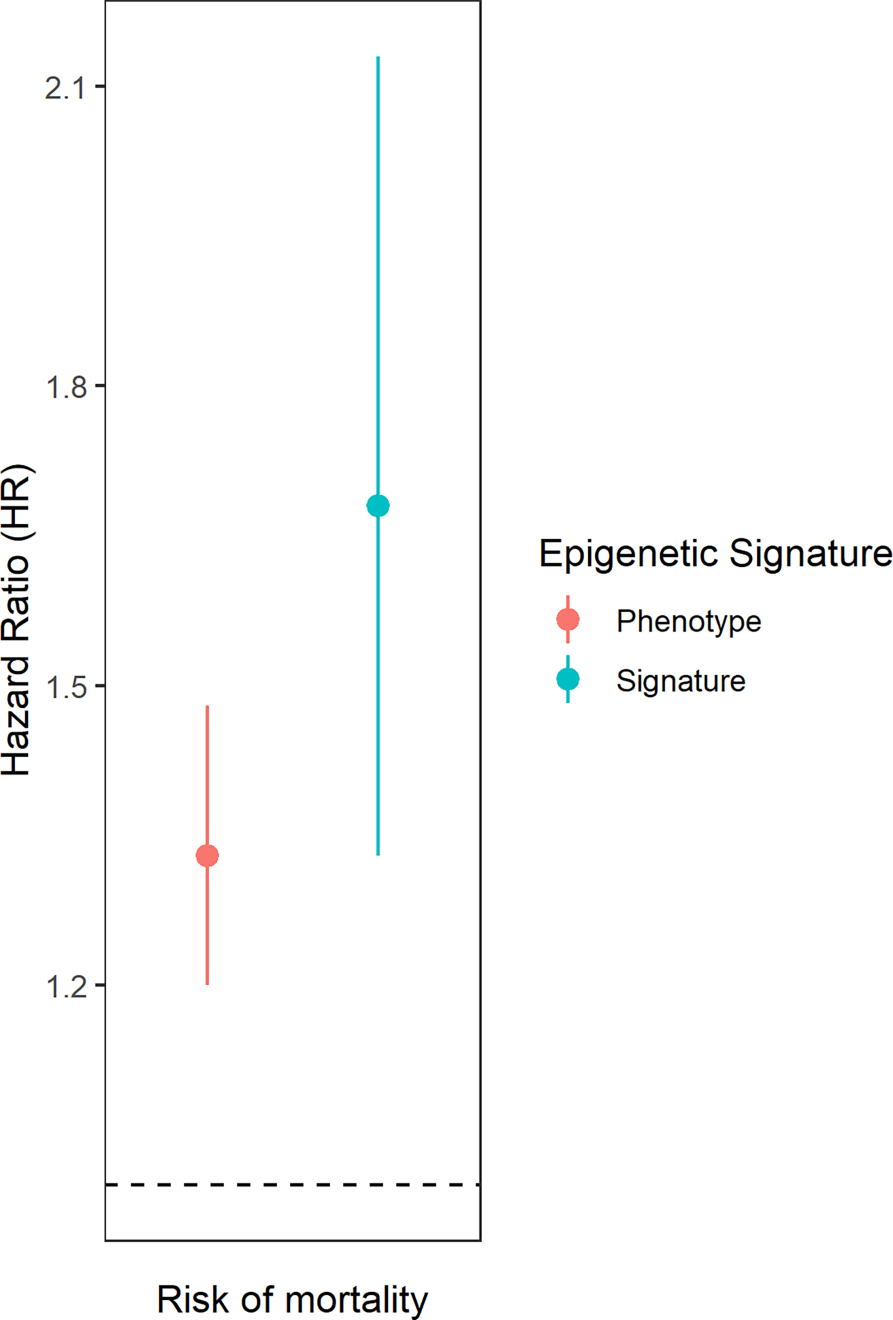
Risk of mortality according to the method used to measure allostatic load in the LBC1936 cohort. Filled-in circles represent the point estimate, while the solid lines correspond to the 95% confidence interval. All presented hazard ratios correspond to the standardized signature/score. LBC1936, Lothian Birth Cohort 1936.

Non-proportional hazards were observed for the methALT signature in relation to all-cause mortality (Figure 3). Globally, elevated values of the methALT signature corresponded to increased mortality risk in the LBC1936 cohort during follow-up. At the beginning of follow-up, each one SD increase in the methALT signature corresponded to a 1.7-fold increase in the mortality rate (HR = 1.68, 95% CI = 1.33–2.13) when adjusting for all potential known confounders. The HR associated with a one SD increase in methALT signature diminished during the follow-up period, reflecting non-proportional hazards (Figure 3). In contrast to the lack of association observed regarding CVD history, the phenotype-based estimate of AL was associated with increased mortality risk (HR = 1.33, 95% CI = 1.20–1.48; adjusting for potential confounders) (Figure 4).

## Discussion

This study describes the creation and validation of an epigenetic signature of AL. Compared with phenotype-based scores of AL, the epigenetic signature of AL (methAL) was associated with both the history of CVD and mortality. The association between the baseline methAL signature and mortality risk did, however, diminish over the study follow-up period.

### Comparison with previous literature

Phenotype-based measures of AL have been consistently associated with CVD and all-cause mortality. Therefore, we replicated analyses using the methAL signature instead of the phenotype-based AL score as a ‘sanity check’ to ensure that the methAL signature demonstrates similar properties. In the present study, elevated AL as measured by the epigenetic signature was associated with a history of CVD and mortality risk. These replicated results are in line with previous publications that used phenotypic measures of AL. For example, a recent systematic review and meta-analysis similarly observed that the risk of all-cause mortality increased with each one-point increase in AL (pooled HR = 1.11, 95% CI = 1.09–1.14) [21]. However, this estimate is slightly lower than that of the present study. With respect to the observed association between AL and CVD, a cross-sectional analysis of data collected in the context of the Boston Puerto Rican Health Study (*N* = 1,116) found that participants with high AL (AL score ≥ 6) had a four-time higher probability of CVD history [22]. When categorizing the methAL signature in the SKIPOGH cohort, compared with low AL (methAL < 1.5), high AL (>3) was associated with a striking ninefold increase in the odds of self-reported history of CVD (OR = 8.43, 95%CI = 3.05–23.27). Similar categorizations of the phenotypic AL score were attenuated (OR = 1.81, 95% CI = 0.95–3.45). A meta-analysis of six studies estimated that high AL was associated with a 31% increased risk for cardiovascular disease-related mortality in comparison with low AL (pooled HR = 1.31, 95% CI = 1.10–1.57), although there was substantial heterogeneity (I^2^ = 91%) with point estimates ranging from 0.99 to 2.24 [21]. Such heterogeneity could be due to variations in cut-off values for categorization, the method used to operationalize AL (e.g. clinical thresholds or distribution-based thresholds), or even the age distribution of the sampled population. Collectively, across studies, it is difficult to draw direct comparisons due to differences in AL operationalization. Adoption of the methAL signature in place of phenotype-based measures of AL could address limitations related to operationalization and comparability and may even be more sensitive than phenotype-based scores at capturing AL at a cellular level.

### Accelerated allostatic aging

Offsetting current health-associated costs for an aging society requires reducing the disease burden that is accelerated by chronic stress and AL. Many initiatives to promote health and well-being focus on optimal functioning maintained throughout the life course until death (i.e. compression of morbidity) [23]. So-called healthy aging, synonymous with the concept of compression of morbidity, focuses on the expansion of health and quality of life [24]. This definition implies different aging trajectories. Normative, or ‘healthy aging’, may be assumed to be devoid of disease, while ‘unhealthy’ aging leads to disease. The AL model theoretically and practically encompasses internal and external stressors (i.e. the exposome) to create a clinical measure rooted in the biopsychosocial model of disease that acknowledges the complex interplay of individual systems and their corresponding biomarkers [25,26]. As such, this allows for both system-specific contributions to AL and more diffused dysregulation of cellular processes.

A synonym to *unhealthy* aging might thus be accelerated aging, whereby dysregulated aging is characterized by a diminution or loss of functional capacity at a system, organ, or cellular level (e.g. telomere attrition, epigenetic alterations, cellular senescence, and genomic instability), as captured by AL [3]. Epigenetic modifications provide the bridge between different levels of an individual’s exposome and may be a result of and contribute to the interplay between individual exposome components and accelerated aging. Other measures such as telomere length [27], mitochondrial DNA (mtDNA) [28], and epigenetic clocks [29] have been previously proposed as potential biomarkers for biological age, as well as AL. However, the focus has been primarily on the HPA axis (hypothalamus–pituitary–adrenal axis, primary mediator of AL). Moreover, although epigenetic clocks may measure allostatic (over)load in part by way of age acceleration or deceleration, estimates of epigenetic age are necessarily correlated with age and thereby aging and, thus, require a definition of normative aging to identify trajectories of non-normal aging. Given its independence from chronological age, the methAL signature does not require a gold standard definition of normative aging.

### MethAL as a target for primary prevention

The reversibility of epigenetic modifications makes them interesting as targets for prevention and therapeutic interventions given their sensitivity to the exposome and exposome-based modifications [30,31]. Lifestyle, environmental, and genetic factors are known modifiers of trajectories of aging, with ample evidence supporting that modifiable lifestyle and environmental exposures are drivers of chronic disease and accelerated aging [32,33]. Importantly, these same exposures probably contribute to elevated AL, although extant literature is limited [34,35]. Prior research is suggestive, if not promising, with regard to the potential to reduce AL through targeted interventions [36,37]. For example, four of the six studies included in a recent scoping review observed significant changes in the levels of AL post-intervention [36]. However, included studies varied in terms of biomarkers and the AL scoring approach used and were limited in terms of sample size. Assuming signature sensitivity to exposome modifications, the methAL signature may help assuage impediments and limitations of prior intervention research and establishment of global benefit. As mentioned previously, one of the main difficulties in establishing AL both clinically and epidemiologically is related to its heterogeneous operationalization [38,39]. In addition, clinical cut-offs for persons 60 years and older may not be relevant for persons younger than 20 years of age, supporting the need for age group-specific distributions for detecting elevated AL in the prodromal period leading to allostatic overload or disease diagnosis.

An epigenetic-based index of AL circumvents these limitations by measuring AL at the molecular level and importantly discarding the need for study-specific or clinical cut-off values. Supporting this, a study that similarly used data from the LBC1936 cohort considered CRP measured using both serum CRP and an epigenetic signature of CRP [40]. Stevenson et al. observed divergent trajectories of serum CRP of both declining and increasing serum CRP levels with increasing age, whereas DNAm-based CRP increased regardless of assigned latent class [40]. Similar results were observed for serum interleukin-6 (IL-6) and a DNAm-based signature for IL-6 [41]. As such, DNAm-based measures of CRP or other AL-related biomarkers could reflect cumulative exposure instead of a more transient snapshot provided by measuring, for example, serum CRP. Moreover, detection levels of inflammatory cytokines are largely dependent on lab-specific thresholds that vary between facilities, suggesting that DNAm-based signatures may serve as proxies that are more reliable. From a prevention perspective, a cumulative measure of AL is likely more precise at identifying individuals with high AL at risk for developing allostatic overload – or disease – due to chronic exposure to exposome stressors. Moreover, in comparison with phenotypic measures of AL, epigenetic signatures may be more sensitive to smaller, molecular-level effects of interventions given that epigenetic regulation probably facilitates the spread of AL across other organs and systems in the body by way of intergenerational epigenetic inheritance and reflects a cellular-level response to exposome triggers and ensuing AL [42]. However, it is also possible that the epigenetic changes measured by the methAL signature are a downstream effect of AL rather than its direct reflection.

From an epidemiological perspective, a valid measure of AL that can be measured retrospectively using biobanked blood samples when clinical and socioeconomic data are not available could be very useful for standardizing future research and permitting investigations into the link between AL and other outcomes when data required for AL construction were not collected. However, from a clinical perspective, the utility of an epigenetic measure of AL is less clear. For example, to address questions of clinical utility, it would be imperative to determine signature plasticity in response to exposome modifications. It would also be necessary to address whether AL should only be considered as a composite measure and not by its individual parts (i.e. cardiovascular system, immune, and neuroendocrine). Regarding signature plasticity, prior evidence showed changes in DNAm of smoking-associated CpGs within the first-year post-smoking cessation, 3 months post-intervention following dietary modifications, and even exercise-induced epigenetic changes as quickly as 60 minutes post-exercise [43–45]. However, methylation plasticity is likely site-dependent in response to direct and indirect pathway activation. Studies that have stratified analyses to identify system-specific associations have generally found limited or no association between lifestyle factors and individual sub-systems of AL. For example, Obosawin et al. observed a marginally significant, but weak, association with the metabolic system, but not with other sub-systems of AL (i.e. immune, cardiovascular, or neuroendocrine systems) [34]. As such, these results support the need for a multisystemic approach to capture global wear-and-tear. Importantly, all system-specific CpGs identified in initial EWASs are captured by the methAL signature. Moreover, the conceptualization of AL as a reflection of both cumulative and systemic dysregulation complicates the development of individual therapeutic interventions, such as senolytics or other senotherapeutics. Future research is needed to determine whether it is possible to tease apart AL as a transient health state and allostatic aging or accelerated aging as a measure of cumulative exposure to chronic stress. Finally, it remains to be determined if methylation changes associated with the accumulation of AL are causative for ageing or reflect ‘wear-and-tear’. Future research is needed that investigates questions of causality, such as whether the epigenetic modifications captured by the methAL signature are causal for associated outcomes like cardiovascular disease.

### Strengths and limitations

This study uses data from two European, predominantly white population-based cohort studies to identify and validate epigenetic signatures of AL; therefore, results are likely generalizable to adult general populations from similar Western populations. A strength of the present study is the employment of operationalization approaches to create the initial phenotype-based AL score considering the diverse methods used to measure AL in prior literature. Moreover, all CpGs were eligible for inclusion in the methAL signature regardless of the EWAS in which they were identified to ensure a more robust DNAm-based signature that potentially captures potential variation in response to the method used to operationalize AL scores. Interestingly, apart from AL operationalized using a summation of quartiles of risk approach, all operationalization methods were represented by CpGs included in the final methAL signature. In addition, for the SKIPOGH cohort on which EWASs were based, all systems were included in the phenotype-based measure of AL (i.e. metabolic, inflammatory, cardiovascular, and neuroendocrine systems). This was not the case for the LBC1936 cohort, which lacked a biomarker for the neuroendocrine system. However, previous literature has found that measurements of AL without a biomarker for the neuroendocrine system were similarly associated with health outcomes [5,21]. Further, the methAL signature was still highly correlated with phenotypic measures of AL in the LBC1936 study population. This also highlights a benefit of using a DNAm-based signature for AL as it would no longer be necessary to collect numerous tests to measure each system-specific biomarker nor would it be necessary to standardize the measurement units across individual tests. In addition, DNAm-based signatures could be practical for large-scale screenings, particularly in resource-limited areas that lack adequate infrastructure.

Nevertheless, there are some potential limitations in the present study. For example, the history of CVD is based on self-report and was not confirmed through medical record review. Assuming nondifferential misclassification, this would attenuate results toward the null. Another limitation is that even though there were discovery and validation sub-samples, results from the EWAS were not validated in an external cohort. It is unlikely though that this has major implications for the validity of the selected CpGs to serve as a proxy for AL as the epigenetic signature of AL was validated in an external cohort. Moreover, while the reversibility of epigenetics may support epigenetic signatures as interesting targets for prevention, it may also be a limitation given the potential variability of epigenetic modifications, contributing to a loss of long-term stability in the face of an unchanging exposome. When comparing the correlation between the phenotypic AL score and methALT signature across DNAm platforms, there are evident platform effects. However, regardless of the platform used, the methALT signature remained well-correlated with AL phenotype (≥0.35). Another potential limitation could be a selectivity in the population analyzed across waves of follow-up (healthy volunteer bias) [46], resulting in the observed reduction in the HR during the LBC1936 follow-up period. It remains unclear whether the declining association between the epigenetic signature and mortality with age is due to potential healthy volunteer bias or reflects a true diminution of effect. Furthermore, the methAL signature is an EPIC-based signature and is, therefore, incompatible with older studies with DNAm data from the HM450K or 27K platforms. To ensure compatibility with older cohorts, a secondary signature was created based on CpGs included on both the HM450K and EPIC platforms. Finally, while the methAL signature may capture influences of social determinants of health from a conceptual perspective, explicit consideration beyond a single measure of AL is necessary to understand the contribution of environmental stressors to overall CVD mortality.

## Conclusion

The epigenetic-based methAL signature serves as a viable proxy for AL and could help assuage issues related to operationalization. Future research should assess its clinical validity and usefulness to inform targeted, individual-level interventions, as well as signature plasticity in response to exposome modifications.

## Supplementary material

Supplementary Table S1

Supplementary Table S2

Supplementary Figure S1

Supplementary Figure S2

Supplementary Figure S3

Supplementary Figure S4

Supplementary Figure S5

Supplementary Figure S6

Supplementary Figure S7

Supplementary Figure S8

## Data Availability

Data for the SKIPOGH cohort are available upon formal request submitted to the SKIPOGH steering committee (https://www.maelstrom-research.org/study/skipogh). Data for both LBC studies are available upon formal request; additional information can be found on the website: https://www.ed.ac.uk/lothian-birth-cohorts/data-access-collaboration.

## Abbreviations

AL: allostatic load
ALAT: alanine transaminase
CRP: C-reactive protein
CVD: Cardiovascular disease
DNAm: Deoxyribonucleic acid (DNA) methylation
HDL: high-density lipoprotein
IFN-γ: interferon gamma
LBC1936: Lothian Birth Cohort 1936
SKIPOGH: Swiss Kidney Project on Genes in Hypertension
