# Supplementary figures and images for: Development and validation of an epigenetic signature of allostatic load
Source: Biosci Rep. 2025 Apr 9;45(4):247–62. doi: 10.1042/BSR20241663 (PMC12203956; doi:10.1042/BSR20241663)

1

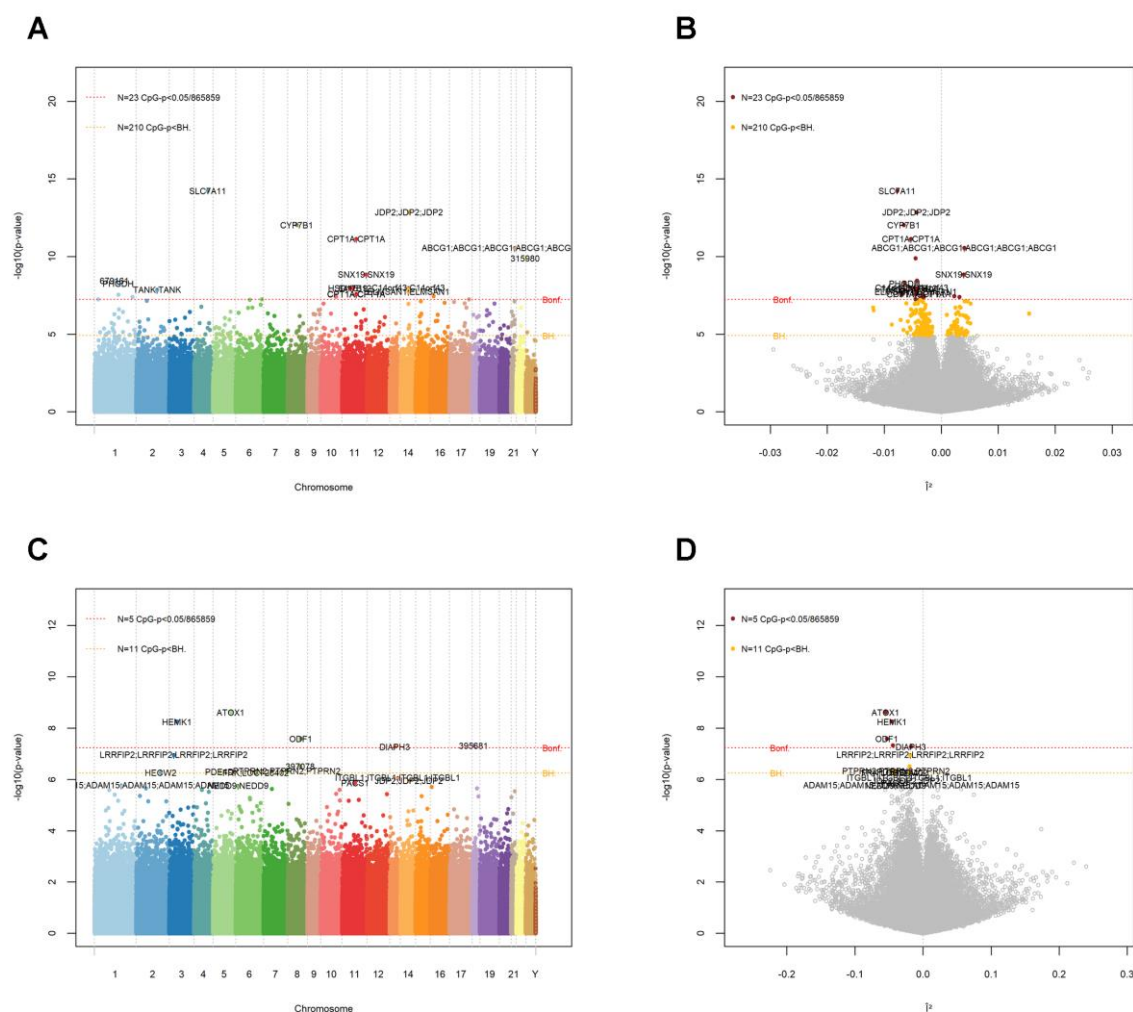

2

3

4

5

6

7

8

9

10

Supplement: Supplementary Figure S1 [file BSR-45-04-BSR20241663-s001.pdf]

Supplementary Figure 4: Latent variable creation for metabolic system

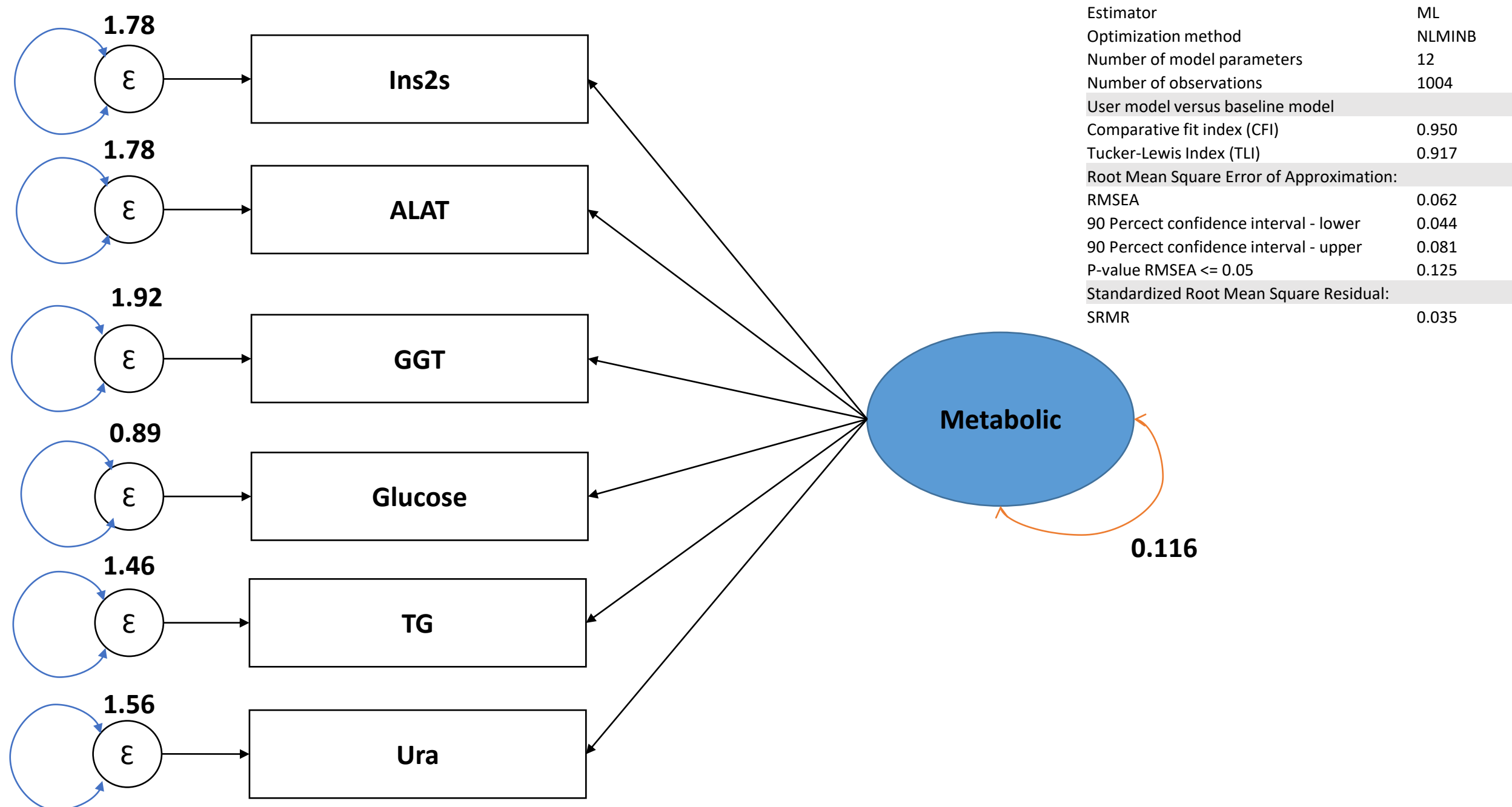

Supplement: Supplementary Figure S4 [file BSR-45-04-BSR20241663-s004.pdf]
