# Supplementary material for: Development and validation of an epigenetic signature of allostatic load
Source: Biosci Rep. 2025 Apr 9;45(4):247–62. doi: 10.1042/BSR20241663 (PMC12203956; doi:10.1042/BSR20241663)

Supplementary Figure 2: Latent variable creation for inflammatory system

Estimator	ML
Optimization method	NLMINB
Number of model parameters	10
Number of observations	1004
User model versus baseline model	
Comparative fit index (CFI)	0.964
Tucker-Lewis Index (TLI)	0.928
Root Mean Square Error of Approximation:	
RMSEA	0.039
90 Percect confidence interval - lower	0.013
90 Percect confidence interval - upper	0.067
P-value RMSEA <= 0.05	0.703
Standardized Root Mean Square Residual:	
SRMR	0.024

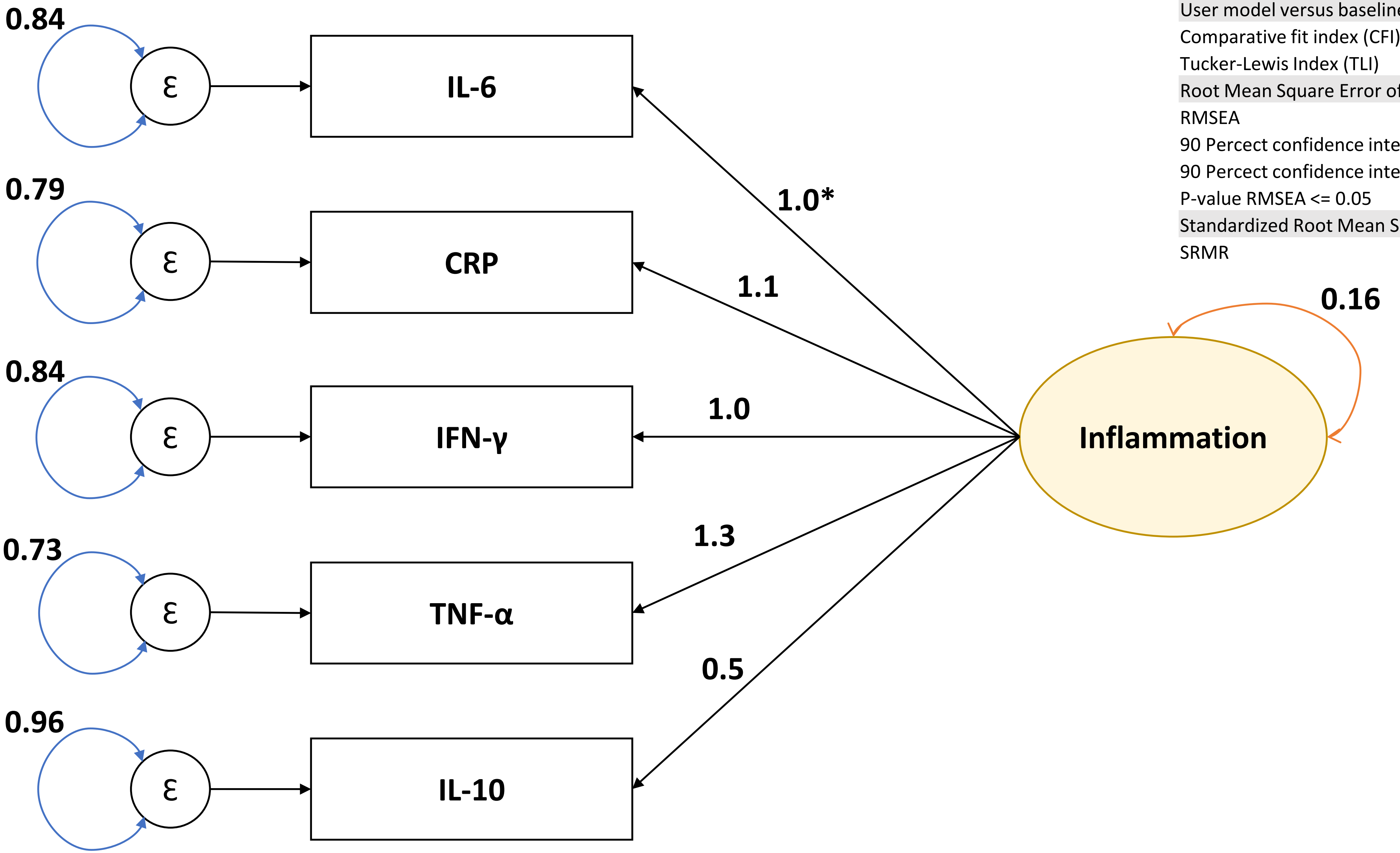

Supplement: Supplementary Figure S2 [file BSR-45-04-BSR20241663-s002.pdf]
