# Supplementary material for: Development and validation of an epigenetic signature of allostatic load
Source: Biosci Rep. 2025 Apr 9;45(4):247–62. doi: 10.1042/BSR20241663 (PMC12203956; doi:10.1042/BSR20241663)
Supplement: Supplementary Figure S3 [file BSR-45-04-BSR20241663-s003.pdf]

Supplementary Figure 3: Latent variable creation for neuroendocrine system

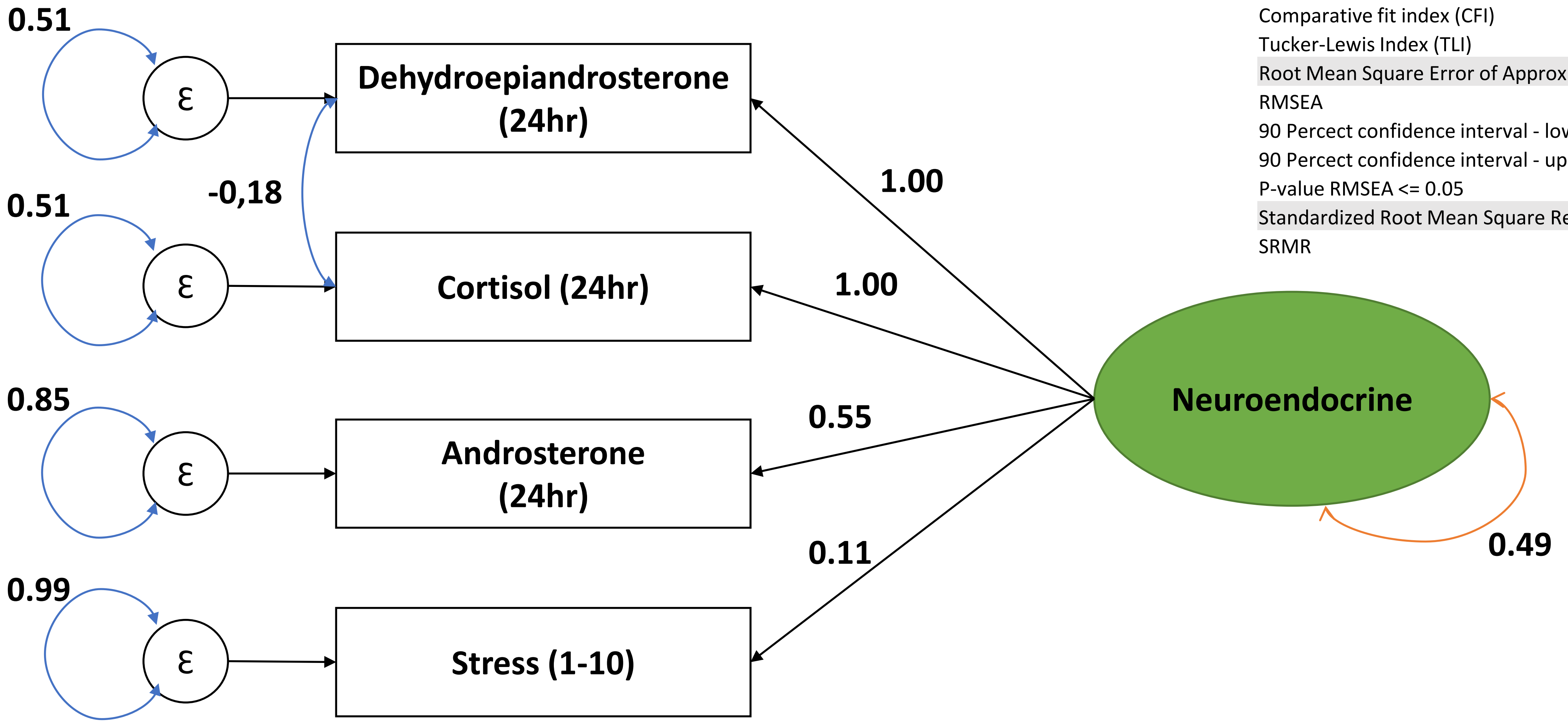

Estimator	ML
Optimization method	NLMINB
Number of model parameters	8
Number of observations	1004
User model versus baseline model	
Comparative fit index (CFI)	0.996
Tucker-Lewis Index (TLI)	0.988
Root Mean Square Error of Approximation:	
RMSEA	0.026
90 Percect confidence interval - lower	0.000
90 Percect confidence interval - upper	0.073
P-value RMSEA <= 0.05	0.747
Standardized Root Mean Square Residual:	
SRMR	0.017
