# Supplementary material for: Development and validation of an epigenetic signature of allostatic load
Source: Biosci Rep. 2025 Apr 9;45(4):247–62. doi: 10.1042/BSR20241663 (PMC12203956; doi:10.1042/BSR20241663)

Supplementary Figure 5: Latent variable creation for cardiovascular system

Estimator	ML
Optimization method	NLMINB
Number of model parameters	5
Number of observations	1004
User model versus baseline model	
Comparative fit index (CFI)	0.974
Tucker-Lewis Index (TLI)	0.921
Root Mean Square Error of Approximation:	
RMSEA	0.131
90 Percect confidence interval - lower	0.083
90 Percect confidence interval - upper	0.186
P-value RMSEA <= 0.05	
Standardized Root Mean Square Residual:	
SRMR	0.032

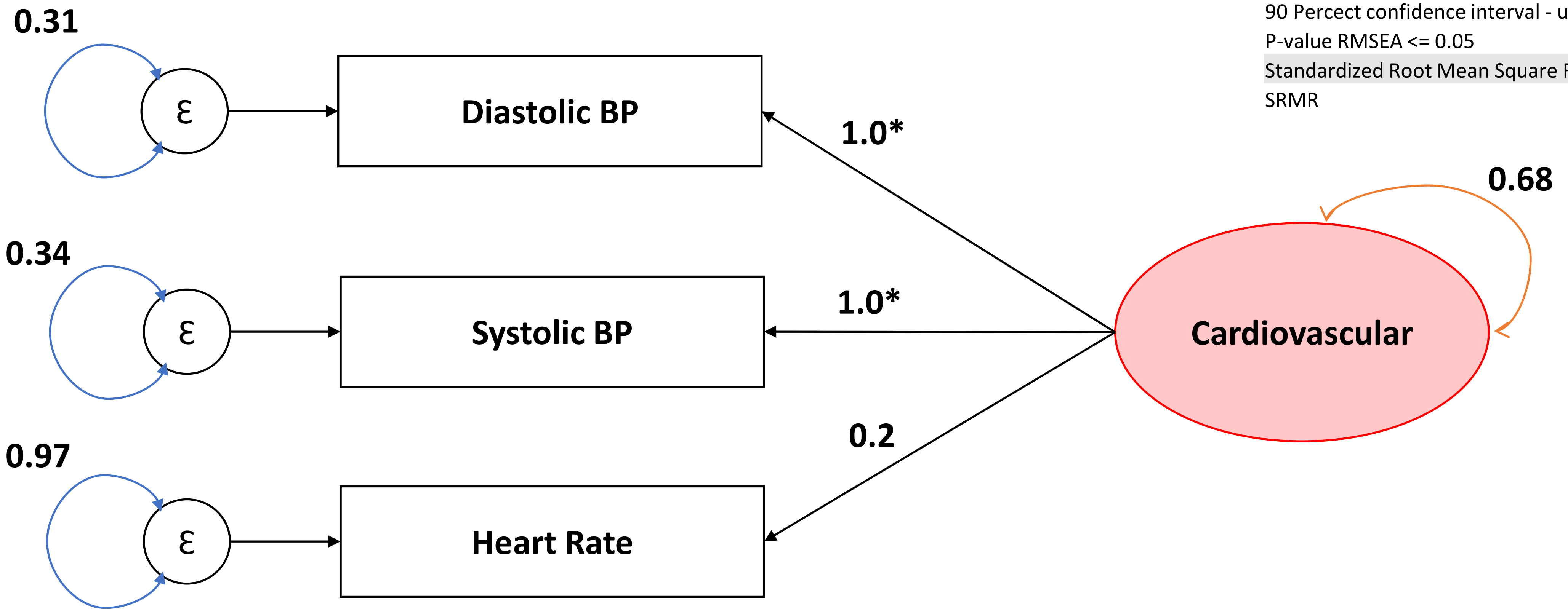

Supplement: Supplementary Figure S5 [file BSR-45-04-BSR20241663-s005.pdf]
