# Supplementary material for: Development and validation of an epigenetic signature of allostatic load
Source: Biosci Rep. 2025 Apr 9;45(4):247–62. doi: 10.1042/BSR20241663 (PMC12203956; doi:10.1042/BSR20241663)
Supplement: Supplementary Figure S6 [file BSR-45-04-BSR20241663-s006.pdf]

Supplementary Figure 6 Volcano and Manhattan plots from discovery EWAS of latent variables

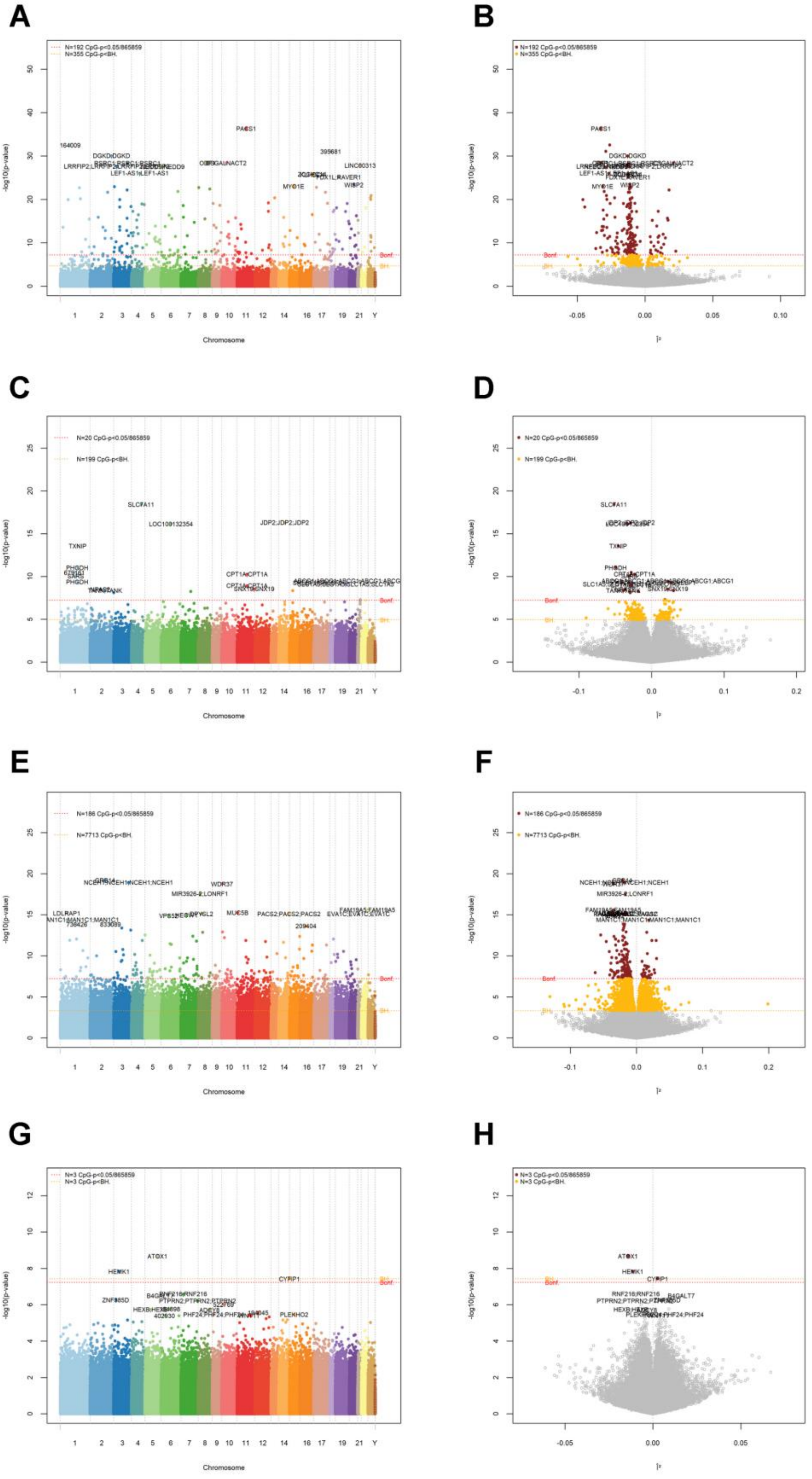

Legend: 6a depicts the Manhattan plot of EWAS results for the latent variable of the neuroendocrine system. 6b depicts the Manhattan plot of EWAS results for the latent variable of the neuroendocrine system. 6c depicts the Manhattan plot of EWAS results for the latent variable of the metabolic system. 6d depicts the Manhattan plot of EWAS results for the latent variable of the metabolic system. 6e depicts the Manhattan plot of EWAS results for the latent variable of the inflammatory system. 6f depicts the Manhattan plot of EWAS results for the latent variable of the inflammatory system. 6g depicts the Manhattan plot of EWAS results for the latent variable of the cardiovascular system. 6h depicts the Manhattan plot of EWAS results for the latent variable of the neuroendocrine system. For each Manhattan plot, individual chromosomes are provided on the x-axis, while the -log10 of the p-value is provided on the y-axis. For the Volcano plots, β values are provided on the x-axis, while the -log10 of the p-value is provided on the y-axis. The yellow dashed line represents the Benjamini-Hochberg adjusted p-value, whereas the red dashed line represents the Bonferroni corrected p-value. Plots A-D were created using data from the SKIPOGH cohort.
