# Supplementary material for: Development and validation of an epigenetic signature of allostatic load
Source: Biosci Rep. 2025 Apr 9;45(4):247–62. doi: 10.1042/BSR20241663 (PMC12203956; doi:10.1042/BSR20241663)

**Supplementary Figure 8** Correlation between the methALT signature and the z-score-based AL score in the LBC1936 cohort

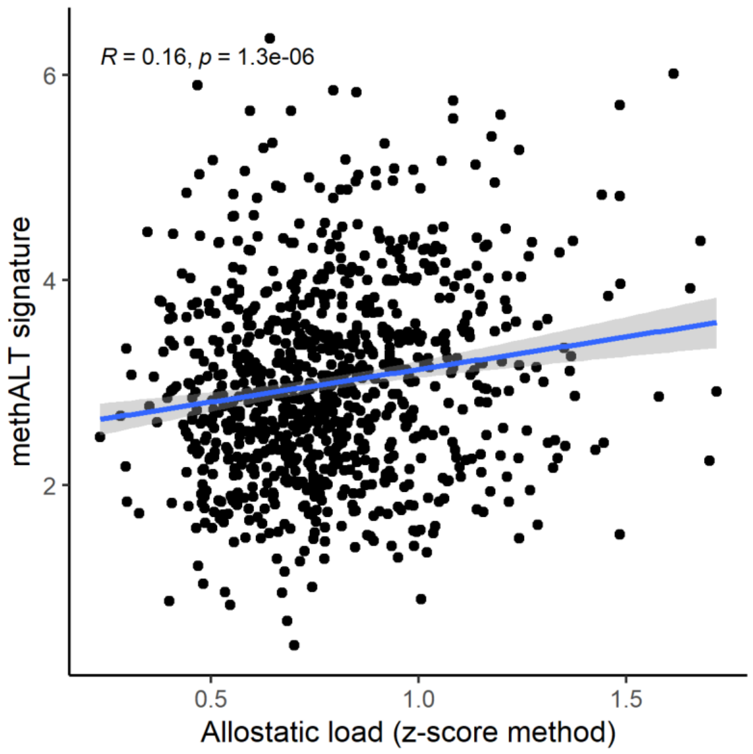

Supplement: Supplementary Figure S8 [file BSR-45-04-BSR20241663-s008.pdf]
